# THE PORCHLIGHT PROJECT: PARTNERING WITH VOLUNTEERS TO ENHANCE COMMUNITY-BASED DEMENTIA CARE AND OUTREACH

**DOI:** 10.1093/geroni/igz038.2927

**Published:** 2019-11-08

**Authors:** Joseph E Gaugler, Christina Rosebush, Gabriela Bustamante, Jeri Schoonover, Roxanne Jenkins, Nicole Bauer, Lisa Beardsley, Laura Rowe

**Affiliations:** 1 University of Minnesota - School of Public Health, Division of Health Policy and Management, Minneapolis, Minnesota, United States; 2 Division of Environmental Health Sciences, School of Public Health, University of Minnesota, Minneapolis, Minnesota, United States; 3 Lutheran Social Service of Minnesota, St. Paul, Minnesota, United States; 4 Lutheran Social Service of Minnesota, Saint Paul, Minnesota, United States

## Abstract

Families often remain unaware of long-term services and supports (LTSS) that could help to mitigate the negative effects of Alzheimer’s disease and related dementias (ADRDs). Approaches that: a) identify community-residing older persons with potential memory impairment; b) assist their families in navigating the healthcare system; and c) facilitate the identification of appropriate community-based LTSS could result in more effective management of ADRD. The Porchlight Project is a multicomponent training approach for lay volunteers in Minnesota (i.e., Senior Companions) that enhances their capability to deliver dementia care and support to underserved older persons in need. Mixed methods analysis of qualitative and quantitative data among 20 Senior Companions and up to 25 persons with ADRD and their family caregivers suggest the potential success of the Porchlight Project, as well as areas to refine and enhance prior to large-scale evaluation throughout Minnesota.

